# Contrast-enhanced ultrasonography as an adjunct to angiography for renal arterial bleeding: a four-case series

**DOI:** 10.1186/s12894-026-02157-7

**Published:** 2026-04-24

**Authors:** Jisun Lee, Yook Kim, Kyung Sik Yi, Chi-Hoon Choi, Bum Sang Cho

**Affiliations:** 1 Department of Radiology, College of Medicine, Chungbuk National University, Chungbuk National University Hospital, 776, 1Sunhwan-ro, Seowon-gu, Cheongju, Republic of Korea; 2 Department of Radiology, Chungbuk National University Hospital, Cheongju, Republic of Korea

**Keywords:** Contrast-enhanced ultrasonography, Renal artery, Pseudoaneurysm, Hemorrhage, Angiography, Embolization, Therapeutic, Kidney injuries

## Abstract

**Background:**

Renal artery bleeding is an uncommon but clinically important complication of surgery, percutaneous procedures, and trauma. Computed tomography (CT) is commonly used; however, indeterminate findings or concerns regarding iodinated contrast can delay hemostasis in some cases. Bedside contrast-enhanced ultrasonography (CEUS) may provide real-time information to support triage to angiography and embolization.

**Case presentation:**

We describe four hemodynamically stable patients: post-nephrectomy, post-percutaneous nephrolithotomy, post-biopsy, and blunt trauma. CT was indeterminate in two cases and deliberately omitted in two cases to avoid delay or contrast exposure. CEUS demonstrated focal contrast pooling, pooling with associated leakage, or jet-like extravasation, helping to localize the suspected culprit branch and supporting subsequent digital subtraction angiography. Superselective coil embolization achieved angiographic stasis in all patients, with no rebleeding during the index admission. Follow-up CEUS at 48 h demonstrated findings consistent with hemostasis, and interval CT showed expected hematoma evolution without residual pseudoaneurysm.

**Conclusion:**

CEUS served as a bedside adjunct decision-support tool across diverse etiologies when CT results were equivocal or deferred, facilitating angiography referral and potentially contributing to timely hemostasis while preserving uninvolved renal parenchyma. This case series demonstrates concordant findings between CEUS and angiography and supports the potential role of CEUS-guided triage for acute renal arterial bleeding.

**Supplementary Information:**

The online version contains supplementary material available at 10.1186/s12894-026-02157-7.

## Background

Renal arterial bleeding is uncommon yet clinically consequential, arising after trauma, surgical or percutaneous interventions (e.g., biopsy, nephrostomy, nephron-sparing surgery), or spontaneous rupture of renal tumors. Rapid recognition and hemostasis are essential to prevent decompensation and preserve renal function. In hemodynamically stable patients, computed tomography (CT) is the first-line imaging modality for evaluating renal arterial bleeding, enabling detection of arterial contrast extravasation, precise localization of the bleeding source, and procedural planning for selective embolization. However, its diagnostic performance may be limited by iodinated-contrast contraindications, renal impairment, artifacts, and indeterminate or low-flow bleeding [1, 2].

Digital subtraction angiography (DSA) provides a confirmatory diagnosis and a platform for transcatheter arterial embolization (TAE), an organ-preserving treatment embedded in contemporary algorithms. However, purely diagnostic DSA without supportive imaging exposes patients to procedural risks, and the diagnostic yield decreases with intermittent or slow-flow bleeding [3, 4].

Contrast-enhanced ultrasonography (CEUS) offers a radiation-free, real-time bedside microvascular assessment using purely intravascular microbubbles and may serve as a complementary tool in clinical decision-making [5]. CEUS may help identify vascular leakage or pseudoaneurysm when CT findings are equivocal and may facilitate clinical decision-making, including triage in blunt trauma, microcirculatory monitoring, and surveillance or re-intervention [6–11].

Despite these advances, reports explicitly positioning CEUS as a decisive trigger for angiography and embolization in acute renal arterial bleeding remain limited. We present four patients across diverse postprocedural scenarios in whom CEUS demonstrated findings suggestive of active bleeding and supported clinical decision-making for subsequent DSA with selective TAE. These cases highlight the potential role of CEUS in facilitating timely endovascular decision-making, shortening the time to hemostasis, and reducing unnecessary additional imaging in acute endovascular care.

## Case series overview

### Diagnostic approach

In this case series, bedside CEUS was used as a problem-solving imaging modality in hemodynamically stable patients with clinically suspected renal arterial bleeding, particularly when contrast-enhanced CT findings were equivocal or when CT was deferred due to concerns regarding iodinated contrast or potential diagnostic delay.

All CEUS examinations were performed using a second-generation blood pool agent (SonoVue; Bracco, Milan, Italy). Findings were interpreted based on dynamic enhancement features such as focal contrast pooling, pooling with associated leakage, and jet-like extravasation [7]. CEUS was also used when rapid bedside assessment was required to guide subsequent angiographic intervention.

### Therapeutic intervention

All procedures were performed using DSA via common femoral arterial access. A 5 F guiding catheter with a coaxial microcatheter system enabled superselective catheterization of the culprit branch, allowing preservation of uninvolved renal parenchyma. Coil embolization was performed using detachable coils (Interlock; Boston Scientific, Natick, MA, USA, and Concerto; Medtronic, Minneapolis, MN, USA), with adjunct gelatin sponge slurry at the operator’s discretion to hasten stasis.

### Follow-up and outcomes

Procedure-related complications were assessed according to the Society of Interventional Radiology classification [12]. Technical and clinical success were observed in all cases, who remained hemodynamically stable without requiring renal replacement therapy. No major changes to the planned embolization strategy were required in any case, and all procedures were well tolerated without significant adverse events beyond those described. Follow-up imaging, when performed, confirmed hemostasis without residual vascular lesions. A summary of patient characteristics, imaging findings, and outcomes is provided in Table 1.

**Table 1:** Consolidated timeline, patient characteristics, and imaging summary

No	Sex/Age (y)	Bleeding etiology	Presentation (symptom/sign; hemodynamics)	Interval^a^ (hrs)	Imaging findings for bleeding			Treatment	Early/interval outcome
					CT	CEUS	Angiography		
1	M / 68	Partial nephrectomy (segmental branch)	POD7: new flank pain; Hb 10.4→7.2; stable	5	Postoperative hematoma; no definite active arterial extravasation across phases	Focal jet-like contrast extravasation adjacent to a segmental arterial branch	Contrast extravasation from a segmental branch	DSA → superselective coil ± gelatin	48-h CEUS: hemostasis; 3-mo CT: no residual vascular lesion
2	F / 72	Percutaneous nephrolithotomy (pseudoaneurysm)	Day 0–1: gross hematuria; Hb 10.4→8.8; stable	14	Not performed^b^	Well-defined round-to-oval contrast-filled sac with arterial communication and to-and-fro behavior, suggestive of pseudoaneurysm	Pseudoaneurysm with a feeding branch	DSA → superselective coil ± gelatin	No rebleeding; discharge stable
3	M / 44	Renal biopsy (vascular injury with extravasation)	Immediate: Doppler suspicious for bleeding; stable	8	Not performed^b^	Focal contrast pooling with associated leakage, suggestive of vascular injury	Contrast extravasation from a lower-pole arterial branch	DSA → superselective coil ± gelatin	48-h CEUS: hemostasis; limited, clinically silent segmental infarct
4	M / 31	Blunt trauma (segmental branch)	Day 0: suspected left renal injury; prior pyeloplasty history; stable	6	High-attenuation material in the left renal pelvis without definite active arterial extravasation; subtle increase in volume on delayed-phase images	Focal jet-like contrast extravasation into the left renal pelvis, suggestive of active bleeding	Contrast extravasation from a segmental renal arterial branch corresponding to the suspected bleeding site	DSA → superselective coil ± gelatin	48-h CEUS: no active bleeding; 1-mo CT: stable

*POD* Postoperative day, *CE* Contrast extravasation (suggestive of active bleeding), *PSA* Pseudoaneurysm, *JP* Jackson–Pratt, *Hb* Hemoglobin, *PCN* Percutaneous nephrostomy, *CEUS* Contrast-enhanced ultrasonography, *CT* Computed tomography, *DSA* Digital subtraction angiography

^a^Interval between the bleeding etiology and CEUS examination

^b^CT was deliberately deferred to avoid treatment delay or iodinated-contrast exposure; bedside CEUS informed angiographic decision-making

## Case presentations

### Case 1

**Fig. 1:**
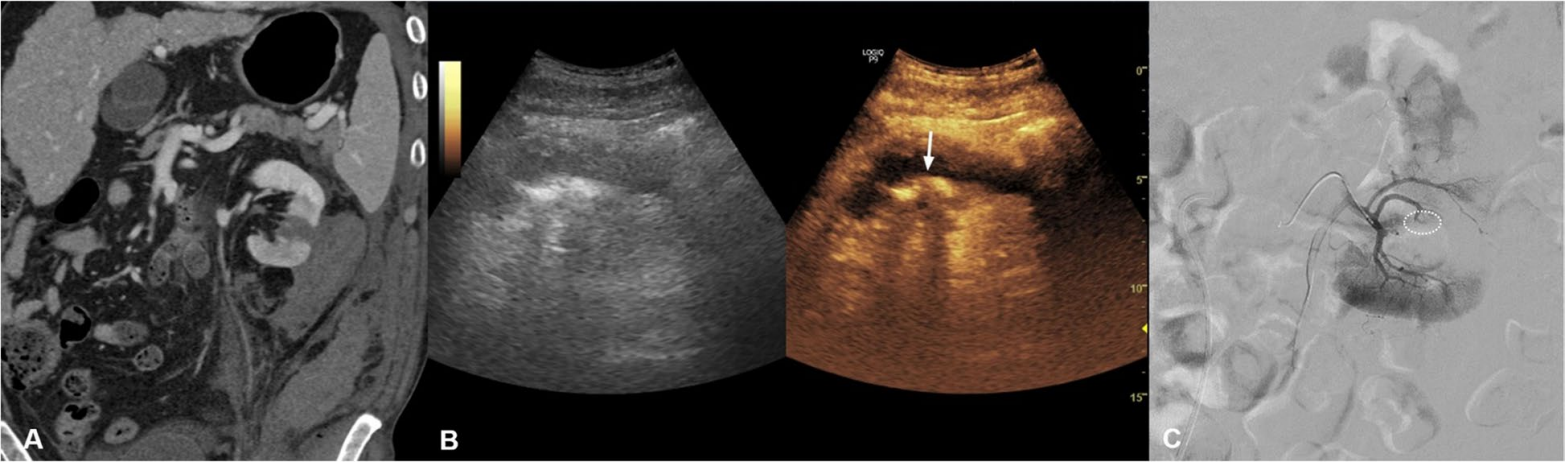
Renal arterial bleeding after partial nephrectomy.** A** Contrast-enhanced CT (portal venous phase, coronal image) demonstrates a postoperative hematoma. A subtle hyperattenuating focus is suspected; however, definite active extravasation is not confidently identified across phases. **B** CEUS demonstrates focal jet-like contrast extravasation adjacent to a segmental branch (arrow), supporting the decision to proceed with angiography for suspected active arterial bleeding. **C** Digital subtraction angiography demonstrates the bleeding focus (circled)

A 68-year-old man underwent left partial nephrectomy for a renal mass confirmed as clear-cell renal cell carcinoma. One week after surgery, he presented with new-onset left flank pain and a decrease in hemoglobin level from 10.4 g/dL to 7.2 g/dL, while remaining hemodynamically stable. Contrast-enhanced CT demonstrated a postoperative hematoma. Although a subtle hyperattenuating focus was suspected, it was not clearly persistent across phases, limiting confident diagnosis. Because persistent bleeding was clinically suspected, bedside CEUS was performed. CEUS demonstrated focal jet-like contrast extravasation adjacent to a segmental arterial branch. DSA demonstrated contrast extravasation from a segmental branch, allowing localization of the bleeding focus. Superselective catheterization was performed, followed by coil embolization using detachable coils (Interlock 3 × 6 cm × 2 and Concerto 3 × 8 cm × 2), with adjunct gelatin sponge. Final angiography demonstrated complete stasis. The patient remained hemodynamically stable without evidence of recurrent bleeding during hospitalization. Follow-up CT at 3 months demonstrated no residual vascular lesion or abnormal findings (Fig. 1).

### Case 2

**Fig. 2:**
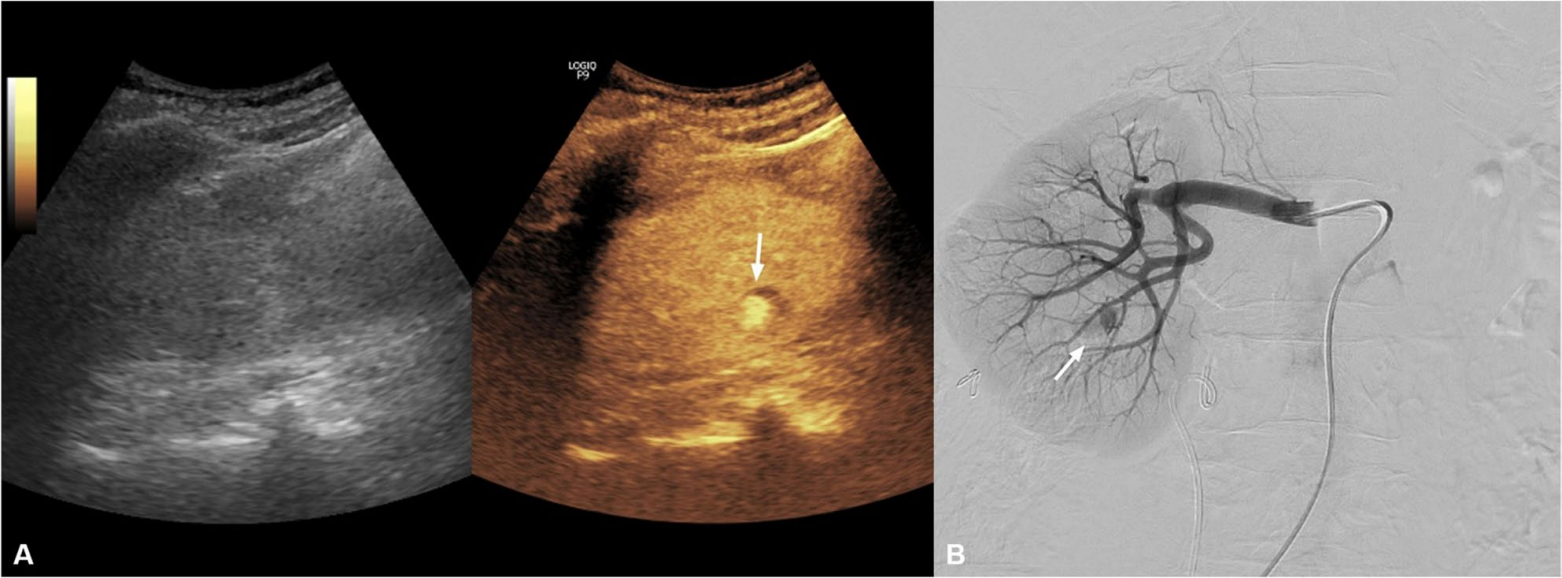
Renal arterial bleeding after percutaneous nephrolithotomy.** A** CEUS demonstrates a well-defined, round-to-oval contrast-filled sac with arterial communication and characteristic to-and-fro flow, consistent with a pseudoaneurysm (arrow). **B** Digital subtraction angiography demonstrates the pseudoaneurysm and its feeding branch (arrow)

A 72-year-old woman with a 3.5-cm right renal stone underwent percutaneous nephrolithotomy. Following the procedure, she developed gross hematuria with a decrease in hemoglobin level from 10.4 g/dL to 8.8 g/dL, while remaining hemodynamically stable. Given the immediate postprocedural setting and strong clinical suspicion of arterial bleeding, bedside CEUS was performed to provide rapid characterization and localization of the suspected vascular lesion prior to angiography, while avoiding potential delay associated with CT. CEUS demonstrated a well-defined, round-to-oval contrast-filled sac with arterial communication and characteristic to-and-fro behavior, consistent with a pseudoaneurysm. Subsequent DSA confirmed the pseudoaneurysm and localized the feeding branch. Superselective catheterization was performed, followed by coil embolization using detachable coils (Interlock 3 × 4 cm × 2 and 3 × 6 cm), with adjunct gelatin sponge. Final angiography demonstrated complete exclusion of the pseudoaneurysm. The patient remained hemodynamically stable without evidence of recurrent hematuria during hospitalization and was discharged in stable condition (Fig. 2).

### Case 3

**Fig. 3:**
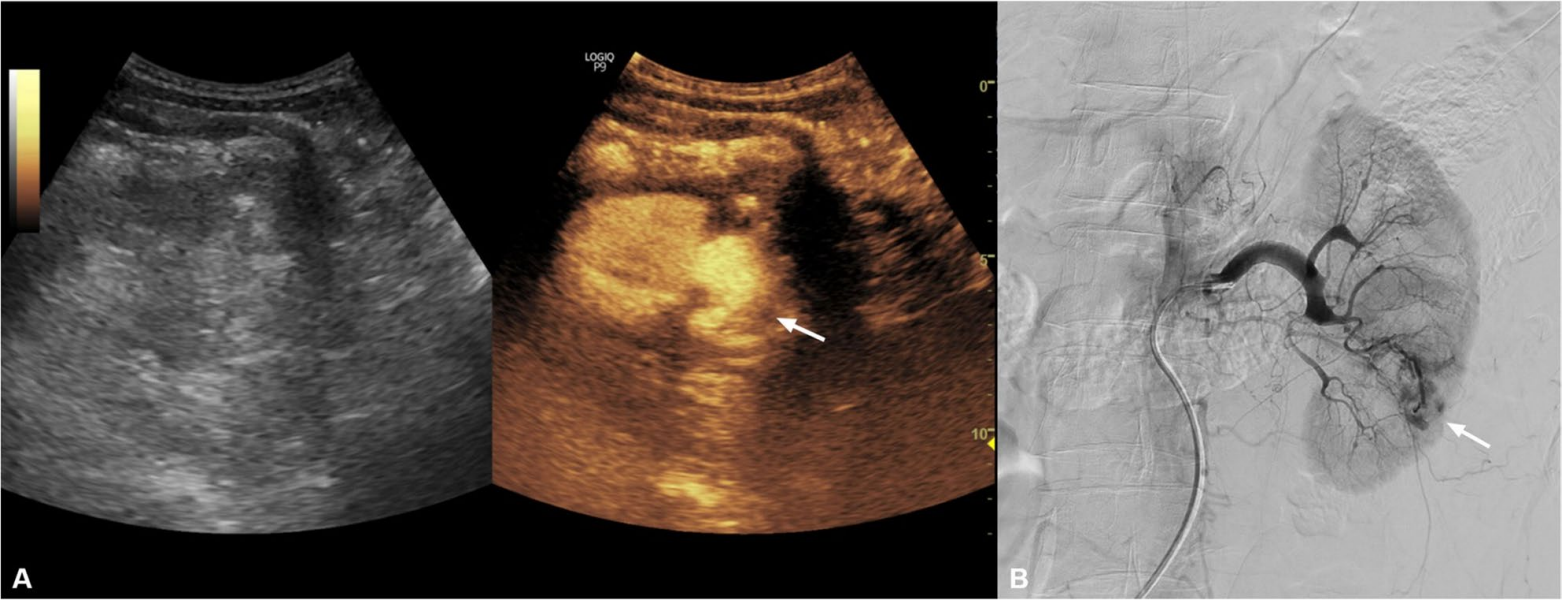
Renal arterial bleeding after renal biopsy.** A** CEUS demonstrates focal contrast pooling with associated leakage communicating with a lower-pole arterial branch, suggestive of vascular injury (arrow). **B** Superselective digital subtraction angiography demonstrates contrast extravasation from a lower-pole arterial branch (arrow)

A 44-year-old man with acute renal dysfunction (serum creatinine 5.17 mg/dL) underwent percutaneous renal biopsy. Immediately after the procedure, color Doppler ultrasound suggested vascular flow suspicious for bleeding. The patient remained hemodynamically stable. Given the impaired renal function and the need for prompt clarification of ongoing bleeding prior to angiography, bedside CEUS was performed as a problem-solving tool to confirm the presence and localization of vascular injury. CEUS demonstrated focal contrast pooling with associated leakage, suggestive of vascular injury. Subsequent DSA demonstrated contrast extravasation from a lower-pole arterial branch. Superselective catheterization was performed, followed by coil embolization using detachable coils (Interlock 2 × 4 cm × 5), with adjunct gelatin sponge. Final angiography demonstrated complete cessation of bleeding. Follow-up CEUS at 48 h confirmed no residual vascular lesion and showed a limited, clinically silent segmental infarct in the treated territory (Fig. 3).

### Case 4

**Fig. 4:**
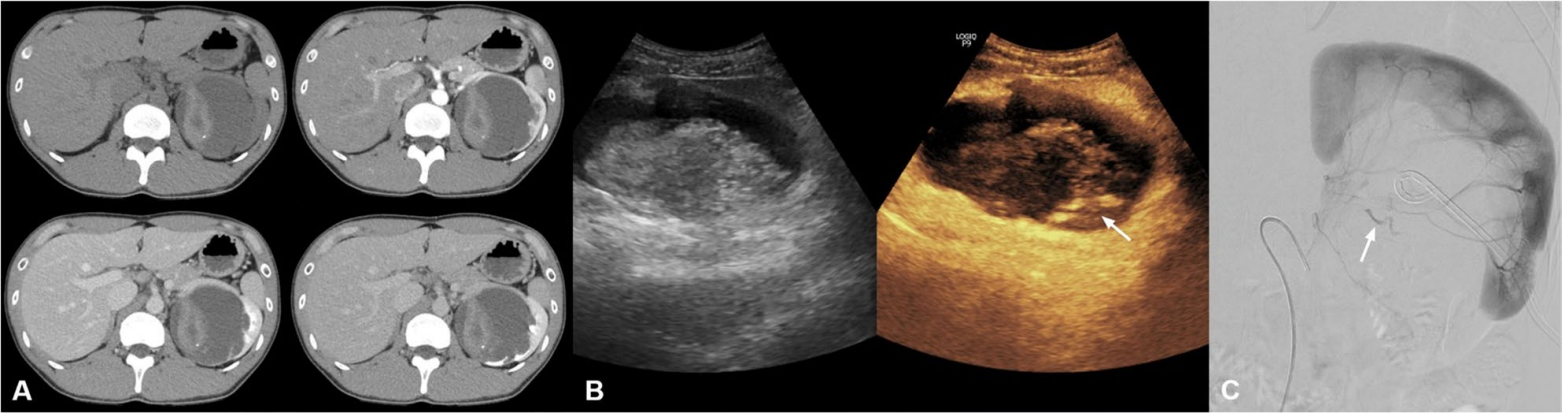
Renal arterial bleeding after blunt trauma.** A** Multiphase contrast-enhanced CT images (pre-contrast, arterial, portal venous, and delayed phases) demonstrate high-attenuation material within the left renal pelvis, consistent with hemorrhage, without definite active arterial extravasation; however, a subtle increase on delayed-phase images raises suspicion for ongoing bleeding. **B** CEUS demonstrates focal jet-like contrast extravasation into the left renal pelvis, suggestive of active bleeding (arrow). **C** Digital subtraction angiography demonstrates active arterial extravasation from a segmental renal arterial branch (arrow)

A 31-year-old man presented with blunt abdominal trauma. He had a remote history of left laparoscopic pyeloplasty for ureteropelvic junction obstruction a decade earlier, without known recurrent obstruction. The patient remained hemodynamically stable. Initial multiphase contrast-enhanced CT, including pre-contrast, arterial, portal venous, and delayed phases, demonstrated high-attenuation material within the left renal pelvis, consistent with hemorrhage but without definite active arterial contrast extravasation. However, a subtle increase in its volume on delayed-phase images raised suspicion for ongoing bleeding. Given persistent clinical concern for arterial bleeding despite inconclusive CT findings, bedside CEUS was performed for further evaluation. CEUS demonstrated focal jet-like contrast extravasation into the left renal pelvis, suggestive of active bleeding. Percutaneous nephrostomy was performed after CEUS and prior to angiography. Subsequent DSA demonstrated contrast extravasation from a segmental renal arterial branch, consistent with the suspected bleeding site. Superselective catheterization was performed, followed by coil embolization using detachable coils (Interlock 2 × 4 cm × 4, 2 × 6 cm × 2, and 3 × 6 cm × 3). Final angiography confirmed complete cessation of bleeding. The patient remained hemodynamically stable without evidence of recurrent bleeding. Follow-up CEUS at 48 h demonstrated no residual contrast extravasation, and one-month CT showed stable postembolization findings without new abnormalities (Fig. 4).

## Discussion

In this four-patient case series including post-nephrectomy, post-percutaneous nephrolithotomy, post-biopsy, and blunt trauma presentations, bedside CEUS provided clinically useful real-time information when contrast-enhanced CT was equivocal or deliberately deferred. While angiography remains the confirmatory standard, CEUS findings helped localize the bleeding source and supported decision-making for triage to DSA, facilitating superselective embolization within the acute endovascular workflow [1].

Contrast-enhanced CT remains the first-line imaging modality in hemodynamically stable patients for global assessment and demonstrating arterial contrast extravasation, which predicts the need for angiography and embolization [1]. When CT is limited by iodinated-contrast contraindications, renal dysfunction, artifacts, or low-flow/intermittent bleeding that yield indeterminate reads, current guidelines generally position CEUS as an adjunct imaging modality in nonhepatic applications, including renal indications for characterization, surveillance, and follow-up [5]. In addition, CEUS is feasible at the bedside, avoids ionizing radiation, and uses intravascular nonnephrotoxic microbubble agents [13]. In practice, CEUS was selectively applied in hemodynamically stable patients when iodinated contrast could not be administered, when renal function was impaired, when rapid confirmation of an arterial source was required prior to intervention, or when clinical suspicion persisted despite indeterminate CT findings [7]. Within this framework, bedside CEUS was used in conjunction with angiography as a confirmatory standard to guide decisions regarding superselective embolization [1, 13].

We used literature-based CEUS terminology as a reporting shorthand [7]. This descriptive approach based on dynamic enhancement features helped clarify indeterminate CT findings by supporting the presence and localization of vascular injury in case 1 and demonstrating focal contrast pooling with associated leakage in case 3. Subsequent angiography demonstrated concordant findings. However, this approach remains operator-dependent and may be influenced by user experience and real-time interpretation, which can limit reproducibility across institutions. In addition, image acquisition and interpretation may vary depending on technical factors such as probe positioning, patient habitus, and acoustic window, further affecting consistency across operators.

In all four cases, CEUS impressions corresponded with angiographic findings and enabled superselective coil embolization, with technical success achieved in each case and no repeat embolization during the index admission period. These outcomes are consistent with contemporary reports demonstrating high clinical success and low complication rates in emergency and postprocedural renal embolization [14, 15]. This concordance suggests that pre-angiographic CEUS localization may help support procedural planning while preserving uninvolved renal parenchyma.

Follow-up imaging highlights the complementary roles of CEUS for early confirmation of hemostasis and CT for subsequent assessment of postembolization changes. In cases where follow-up imaging was performed, 48-hour CEUS demonstrated findings consistent with hemostasis, and subsequent CT demonstrated stable postembolization changes without new pathology. Previous reports have also described the use of CEUS in posttreatment surveillance of renal arterial lesions, including confirmation of hemostasis and detection of small residual flow in follow-up settings [10, 11].

This case series has several limitations. Although the number of cases is small, the included cases represent clinically relevant scenarios in which conventional imaging alone was insufficient to guide management. The single-center design and nonstandardized selection of CEUS reflect real-world practice rather than a predefined diagnostic protocol. In addition, CEUS is inherently operator-dependent, and its acquisition and interpretation may vary with user experience and technical factors such as probe positioning, patient habitus, and acoustic window, which may limit reproducibility. Furthermore, the absence of standardized criteria for CEUS application and the limited number of cases restrict the generalizability of these observations to broader clinical settings.

From a clinical perspective, CEUS may serve as an adjunct imaging modality in selected hemodynamically stable patients with suspected renal arterial bleeding. It may be particularly useful when CT findings are inconclusive, when iodinated contrast cannot be administered, or when rapid confirmation of a vascular source is required to guide endovascular management. In such scenarios, bedside CEUS can provide real-time information that supports clinical decision-making and may support timely referral for angiographic intervention. These observations suggest that CEUS may play a complementary role in bridging indeterminate or deferred CT findings and definitive angiographic treatment in carefully selected clinical settings. In this context, contrast-enhanced CT remains the primary modality for global evaluation in stable trauma [1].

In this four-patient case series, CEUS findings were concordant with angiography and supported decision-making for embolization. These observations suggest a complementary adjunct role for CEUS in selected hemodynamically stable patients with suspected renal arterial bleeding.

## Supplementary Information

Below is the link to the electronic supplementary material.

Supplementary Material 1.

Supplementary Material 2.

## Data Availability

The data supporting the findings of this case series are not publicly available due to the inclusion of potentially identifiable clinical information but are available from the corresponding author upon reasonable request.

## Abbreviations

CEUS: Contrast-enhanced ultrasonography
CT: Computed tomography
DSA: Digital subtraction angiography
TAE: Transcatheter arterial embolization
Hb: Hemoglobin
POD: Postoperative day
PSA: Pseudoaneurysm
JP: Jackson–Pratt
PCN: Percutaneous nephrostomy
