# Supplementary material for: Contrast-enhanced ultrasonography as an adjunct to angiography for renal arterial bleeding: a four-case series
Source: BMC Urol. 2026 Apr 24;26:137. doi: 10.1186/s12894-026-02157-7 (PMC13231711; doi:10.1186/s12894-026-02157-7)
Supplement: Supplementary file 1 — Supplementary Material 1. [file 12894_2026_2157_MOESM1_ESM.docx]

**Supplementary Table 1. Detailed clinical characteristics and procedural timeline of the four patients**

| **No** | **Comorbidities** | **Antiplatelet / Anticoagulant status** | **Baseline Serum Creatinine (mg/dL)**  **[eGFR (mL/min/1.73 m²)]** | **Post-embolization Serum Creatinine (mg/dL)**  **[eGFR (mL/min/1.73 m²)]** | **Time from CEUS to DSA (minutes)** | **Follow-up Duration (months)** | **Clinical Follow-up Outcome** |
| --- | --- | --- | --- | --- | --- | --- | --- |
| 1 | History of gastric cancer surgery  (10 years prior) | No antiplatelet or anticoagulant therapy | 0.92 [88.0] | 0.79 [105.0] | 30 | 48 | Clinically stable without recurrence |
| 2 | Hypertension; diabetes mellitus | No antiplatelet or anticoagulant therapy | 0.60 [92.4] | 0.72 [84.8] | 20 | 36 | Clinically stable without recurrence |
| 3 | Hypertension; chronic kidney disease (biopsy-proven hypertensive nephrosclerosis) | No antiplatelet or anticoagulant therapy | 5.17 [12.6] | 5.20 [12.5] | 20 | 6 | Clinically stable without recurrence |
| 4 | History of left laparoscopic pyeloplasty for ureteropelvic junction obstruction (10 years prior) | No antiplatelet or anticoagulant therapy | 1.32 [71.4] | 1.15 [84.3] | 90 | 12 | Clinically stable without recurrence |

CEUS, contrast-enhanced ultrasonography; DSA, digital subtraction angiography; eGFR, estimated glomerular filtration rate.
