# Supplementary material for: Contrast-enhanced ultrasonography as an adjunct to angiography for renal arterial bleeding: a four-case series
Source: BMC Urol. 2026 Apr 24;26:137. doi: 10.1186/s12894-026-02157-7 (PMC13231711; doi:10.1186/s12894-026-02157-7)
Supplement: Supplementary file 2 — Supplementary Material 2. [file 12894_2026_2157_MOESM2_ESM.pdf]

CARE Checklist of information to include when writing a case report

Topic	Item	Checklist item description	Reported on Line(s)
Title	1	The diagnosis or intervention of primary focus followed by the words 'case report'	Lines 1–2
Key Words	2	2 to 5 key words that identify diagnoses or interventions in this case report, including 'case report'	Lines 17–18
Abstract	3a	Introduction: What is unique about this case and what does it add to the scientific literature?	Lines 21–25
Abstract	3b	Main symptoms and/or important clinical findings	Lines 26–28
Abstract	3c	The main diagnoses, therapeutic interventions, and outcomes	Lines 28–34
Abstract	3d	Conclusion—What is the main take-away lesson(s) from this case?	Lines 35–39
Introduction	4	One or two paragraphs summarizing why this case is unique	Lines 56–68
Patient Information	5a	De-identified patient specific information	Lines 97–101, 113–116, 129–132, 143–146

Patient Information	5b	Primary concerns and symptoms	Lines 99–101, 114–116, 130–132, 143–146
Patient Information	5c	Medical, family, and psycho-social history including relevant genetic information	Supplementary Table 1 (Page 11)
Patient Information	5d	Relevant past interventions with outcomes	Lines 97–99, 113–114, 129–130, 143–144
Clinical Findings	6	Describe significant physical examination and important clinical findings	Lines 99–101, 114–116, 130–132, 143–146
Timeline	7	Historical and current information from this episode of care organized as a timeline	Table 1 (Page 9)
Diagnostic Assessment	8a	Diagnostic testing	Lines 72–80, 101–104, 117–121, 133–136, 146–150
Diagnostic Assessment	8b	Diagnostic challenges	Lines 72–75, 101–103, 146–150
Diagnostic Assessment	8c	Diagnosis (including other diagnoses considered)	Lines 104–106, 120–122, 135–137, 152–155
Diagnostic Assessment	8d	Prognosis where applicable	Supplementary Table 1 (Page 11) and Lines 88–94
Therapeutic Intervention	9a	Types of therapeutic intervention	Lines 81–87
Therapeutic Intervention	9b	Administration of therapeutic intervention	Lines 81–87, 106–108, 123–124, 137–139, 156–157
Therapeutic Intervention	9c	Changes in therapeutic intervention	Lines 91–92

Follow-up and Outcomes	10a	Clinician and patient-assessed outcomes	Lines 88–94, 109–111, 125–127, 139–141, 158–161
Follow-up and Outcomes	10b	Important follow-up diagnostic and other test results	Lines 93–94, 110–111, 140–141, 159–160
Follow-up and Outcomes	10c	Intervention adherence and tolerability	Lines 92–93
Follow-up and Outcomes	10d	Adverse and unanticipated events	Lines 89–90, 92–93
Discussion	11a	Scientific discussion of strengths and limitations	Lines 215–223
Discussion	11b	Discussion of relevant medical literature	Lines 178–186, 201–207
Discussion	11c	Scientific rationale for conclusions	Lines 171–177, 192–194
Discussion	11d	Primary take-away lessons	Lines 224–237
Patient Perspective	12	Patient perspective on treatment	Patient perspectives were not available due to the retrospective nature of this case series.
Informed Consent	13	Did the patient give informed consent?	Lines 256–258
